# Relationship between mean volume voided and incontinence in children with overactive bladder treated with solifenacin: post hoc analysis of a phase 3 randomised clinical trial

**DOI:** 10.1007/s00431-020-03635-2

**Published:** 2020-04-01

**Authors:** Robert Snijder, Brigitte Bosman, Otto Stroosma, Marja Agema

**Affiliations:** Astellas Pharma Europe B.V., Leiden, Netherlands

**Keywords:** Mean volume voided, Incontinence, Overactive bladder, Children, Solifenacin

## Abstract

This post hoc Poisson regression analysis investigated the relationship between mean volume voided and incontinence episodes/24 h after fixed frequency adjustment in children with overactive bladder from the LION study, a phase 3, double-blind, randomised, placebo-controlled, sequential, dose-titration solifenacin trial. Patients were aged 5–< 12 years with ≥ 4 episodes of daytime incontinence during a 7-day pre-baseline diary period. The dependent variable was the mean number of incontinence episodes/24 h at the end of study. Explanatory variables included treatment, mean number of incontinence episodes/24 h at baseline, and change from baseline to end of study in mean volume voided. Statistical significance and goodness of fit were analysed using the Pearson’s chi-square test. A negative estimate was found between the dependent variable ‘incontinence’ and both mean volume voided and daytime maximum volume voided/micturition (an increase in mean volume voided or daytime maximum volume voided/micturition would lead to a reduction in incontinence; *P* = 0.0014 and *P* = 0.0317, respectively). The model was a good fit to the data in both analyses with a Pearson’s chi-square goodness-of-fit criteria of 0.8.

*Conclusion*: Increase in mean volume voided was significantly correlated to reduction in incontinence episodes/24 h in children with overactive bladder treated with solifenacin.

*This study is registered at* *ClinicalTrials.gov**:* NCT01565707.

**Table:** 

**What is known:** *• Mean volume voided per micturition is used as an indicator of treatment efficacy, with increases noted as number of incontinence episodes (and micturition frequency) decrease.* *• The relationship between mean volume voided and incontinence episodes is not clearly understood.* **What is new:** *• Increase in mean volume voided significantly correlated to reduction in incontinence in solifenacin-treated children with overactive bladder (Poisson regression model analysis).* *• Compared with placebo, solifenacin-treated children had a lower predicted number of incontinence episodes/24 h.*

**What is known:**

*• Mean volume voided per micturition is used as an indicator of treatment efficacy, with increases noted as number of incontinence episodes (and micturition frequency) decrease.*

*• The relationship between mean volume voided and incontinence episodes is not clearly understood.*

**What is new:**

*• Increase in mean volume voided significantly correlated to reduction in incontinence in solifenacin-treated children with overactive bladder (Poisson regression model analysis).*

*• Compared with placebo, solifenacin-treated children had a lower predicted number of incontinence episodes/24 h.*

## Introduction

Overactive bladder (OAB) syndrome is defined by the International Children’s Continence Society as urinary urgency, usually accompanied by frequency and nocturia with/without urinary incontinence, in the absence of urinary tract infection or other obvious pathology [1]. A potential major drawback of this definition is the oversimplification of multifactorial symptoms to indicate that OAB is a uniform clinical entity [2–4]. Moreover, OAB is defined by the subjective symptom of urgency and requires no other diagnostic symptom or urodynamic assessment. However, an increase in urgency leads to increased frequency/incontinence resulting in reduced bladder capacity, which is often associated with OAB.

In clinical studies of OAB, mean volume voided (MVV)/micturition is widely used as an objective and physiological indicator of treatment efficacy [5–8]. MVV refers to the mean volume of voided urine measured on the frequency volume chart throughout a 24-h cycle, and several studies have suggested an association between MVV and OAB symptoms. Antimuscarinics are commonly used as first-line pharmacotherapies to treat OAB symptoms in adults after conservative treatment has failed [9]. In a meta-analysis of placebo response in antimuscarinic trials for OAB, focusing on adults, change in MVV was negatively associated with changes in incontinence episodes and micturitions/day [10]. Additionally, in clinical trials, treatment with the antimuscarinic, solifenacin, was associated with increases in MVV and reductions in incontinence episodes in adults with OAB [7, 11–13]. However, the relationship between MVV and incontinence is not clearly understood, especially in paediatric patients.

Newgreen et al. previously reported results from a phase 3, randomised, placebo-controlled study, which evaluated the efficacy and safety of solifenacin in children (aged 5–< 12 years) and adolescents (aged 12–< 18 years) with OAB (LION study) [14]. The primary efficacy endpoint was MVV/micturition and secondary endpoints included daytime maximum volume voided/micturition (DMaxVV), mean incontinence episodes/24 h, mean number of incontinence-free days or nights/7 days, and micturition frequency. In children, solifenacin was superior to placebo in changes from baseline to end of treatment (EoT) in MVV/micturition, DMaxVV, and micturition frequency adjusted for baseline total voided volume. Solifenacin was well tolerated in both children and adolescents. Here we report a post hoc analysis of the efficacy data from LION to investigate the relationship between MVV and incontinence episodes/24 h after fixed frequency adjustment in children.

## Methods

LION was a phase 3, double-blind, randomised, placebo-controlled, sequential, dose-titration study conducted in children aged 5–< 12 years and adolescents aged 12–< 18 years, who had been diagnosed with OAB according to the International Children’s Continence Society criteria, and who had ≥ 4 episodes of daytime incontinence during a 7-day pre-baseline diary period. The study was conducted in accordance with the ethical principles of the Declaration of Helsinki, Good Clinical Practice, International Conference on Harmonisation guidelines, and applicable laws and regulations. An independent ethics committee/institutional review board for each site approved the study before initiation. Informed consent was provided by the patients’ parent(s)/legal representative(s), and, where appropriate, the patient provided written assent. Patients underwent a 4-week urotherapy run-in period (including a timed schedule of seven voids/24 h). After 2 weeks, single-blind placebo administration was combined with urotherapy. At baseline, patients were randomised 1:1 by country to receive double-blind solifenacin oral suspension or placebo once daily for 12 weeks alongside urotherapy. The primary findings of the study and the methodology have been published elsewhere [14].

To analyse the relationship between MVV and incontinence episodes/24 h, Poisson regression was performed using the SAS GENMOD procedure. The dependent variable was the mean number of incontinence episodes/24 h at the end of study (EoS). Explanatory variables included treatment, mean number incontinence episodes/24 h at baseline, and change from baseline to EoS in MVV. The Pearson chi-square test was used to analyse statistical significance and goodness of fit; a model with a good fit would have a goodness-of-fit statistic close to or below one.

## Results

In total, 148 children aged 5–< 12 years were randomised to receive urotherapy plus solifenacin (*n* = 73) or placebo (*n* = 75). Overall, 54.1% of children (60.3% solifenacin, 47.9% placebo) were female (mean age, 7.5 years).

**Fig. 1:**
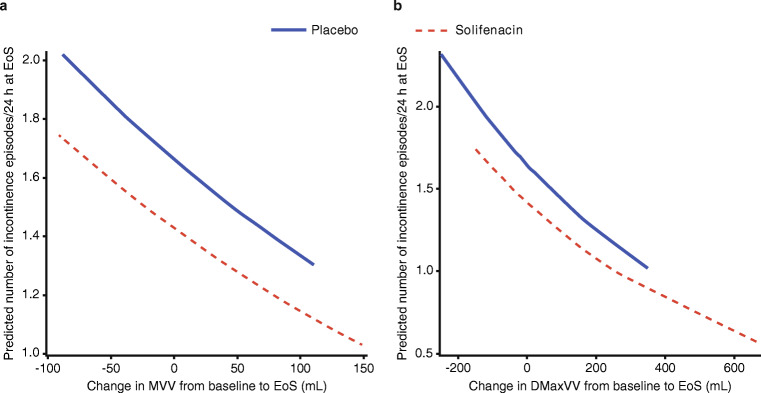
Validity of Poisson regression model with MVV/micturition **a** or DMaxVV **b** as predictors of incontinence. Response Variable (Y-axis) represents the predicted number of incontinence episodes/24 h at EoS. Predictor Variable (X-axis) represents the change from baseline to EoS in MVV **a** or DMaxVV **b**

From the Poisson regression analysis, a negative estimate was found for the relationship between the dependent variable ‘incontinence’ and MVV (an increase in MVV will lead to a reduction in incontinence). This estimate was statistically significant (*P* = 0.0014). Pearson’s chi-square criteria for goodness of fit of 0.8 indicated that the model was a good fit to the data (Table 1 and Fig. 1a).

**Table 1:** Analysis of mean number of incontinence episodes/24 h^a^

	Placebo (*n* = 89)	Solifenacin (*n* = 94)	Total (*n* = 183)
*n*	70	73	143
Incontinence episodes/24 h, mean (SD)			
Baseline	2.98 (2.6)	2.46 (2.6)	2.71 (2.6)
EoS	1.63 (1.4)	1.47 (1.2)	1.55 (1.3)
Poisson model			
*P* value for change from baseline in MVV		0.001	
Goodness of fit^b^		0.823	
*P* value for change from baseline in DMaxVV		0.024	
Goodness of fit^b^		0.836	

^a^Change from baseline in MVV/micturition and DMaxVV were used as basic predictors

^b^Statistic close to or below one indicates a good fit

A Poisson regression analysis with DMaxVV as predictor indicated a statistically significant negative relationship between DMaxVV and incontinence (Fig. 1b). The relationship was less prominent compared with the relationship between MVV and incontinence (*P* = 0.0317); Pearson’s chi-square criteria for goodness of fit for this model was 0.8.

## Discussion

In this study, we previously found that solifenacin-treated children (5–< 12 years) with OAB had a greater change from baseline to EoT in MVV (primary endpoint) than placebo-treated children [14]. The current analysis showed that increases in MVV statistically predicted reductions in incontinence episodes/24 h in this patient population. Compared with placebo, solifenacin-treated children had a lower predicted number of incontinence episodes/24 h, consistent with previous studies in adults [7, 11–13]. Importantly, we observed this correlation with a small population of 143 children, which indicates the clinical relevance of our finding.

Poisson regression provides an appropriate framework for modelling incontinence episodes and other count data [15]. Solifenacin treatment reduced incontinence episodes to close to zero by EoT. In cases where the outcome variable has a low arithmetic mean, the use of standard ordinary least squares regression may introduce bias. Moreover, a Poisson model permits the accommodation of overdispersion, an inherent problem of count data.

Correlations between commonly used efficacy endpoints have been previously investigated in adults with OAB [7, 10–13]. Our study results increase the body of evidence that suggests these correlations also exist in children. When incontinence/frequency increases in severity, bladder filling until voiding or involuntary loss of urine will be reduced, decreasing MVV. Hence, correlations between MVV and incontinence or micturition frequency would be expected. Although our study design did not enable us to evaluate correlations between frequency and MVV, a retrospective study of 548 children who presented with urgency demonstrated that in > 90% of children, urgency was accompanied by either daytime incontinence (72.6%, more common in girls) and/or frequency (48.9%, more common in boys) [16]. Expected bladder capacity decreased as the number of symptoms, in addition to urgency, increased. Chiang et al. reported significant correlations between first morning voided volume and MVV or cystometric capacity [17]. In addition, a review of randomised clinical trials showed an inverse correlation between MVV/micturition and micturition frequency/24 h [18]. Similarly, a negative association between daytime MVV and nocturia was reported in patients with lower urinary tract symptoms [19]. Finally, van Brummen et al. showed that frequency was associated with objective parameters from the bladder diary, filling cystometry, and detrusor overactivity [20].

In LION, MVV was selected as the primary endpoint because it is the most reproducible of the quantitative OAB treatment endpoints in adults with low inter-patient and intra-patient variations. MVV is a recognised and recommended endpoint by European authorities for paediatric studies [14, 21]. Moreover, in clinical trials, MVV increases have been used as indicators of successful OAB treatment in adults [5–8]. In LION, the increase in MVV observed in solifenacin-treated children versus placebo was considered likely to be clinically relevant [14]. Given its importance as a primary endpoint, we suggest the inclusion of MVV in the OAB definition.

The reduction from baseline in the number of incontinence episodes at EoT was not significantly different between the solifenacin and placebo arms [14]. A potential factor responsible could be the small sample size. To power the study to detect statistical significance in incontinence or micturition frequency, the required patient number would have needed to increase to 520 per treatment, too large for a phase 3 study in paediatric patients with OAB. A small sample size may also result in a higher risk of imbalance between the treatment groups for clinically relevant baseline characteristics. A similar lack of treatment effect on incontinence associated with a small sample size has been previously reported in a phase 2 study of solifenacin in adults [22].

A large placebo response was observed for the incontinence endpoint in children, similar to previous studies in adults treated with solifenacin [7, 11]. This could be attributed to the measures implemented for managing OAB symptoms including urotherapy, use of bladder diaries, and increased ability of children to control their bladder function as age increases. Additionally, there was an imbalance in the number of incontinence episodes at baseline. Patients were required to have ≥ 4 incontinence episodes in the 7-day period before the baseline visit. However, during randomisation, patients were not stratified according to their degree of incontinence, and the mean number of incontinence episodes/24 h was higher in the placebo group. In placebo-treated patients from several OAB studies, a positive correlation between baseline severity and change in frequency of incontinence episodes has been observed [10].

Adolescent recruitment into the LION trial was challenging given the lower prevalence of OAB in adolescents than in children and the difficulty of enrolling adolescents in controlled clinical trials [23–26]. As 41 out of the planned target of ≥ 120 adolescents were enrolled, statistical power was reduced, and no conclusions could be drawn for the primary and secondary efficacy variables. Hence, this analysis did not include the adolescents enrolled in the trial. Study limitations also included the inherent problems associated with post hoc analyses. The study was based on a hypothesis that needs to be validated in a prospective clinical study involving children with OAB.

In conclusion, our study demonstrated that MVV increases significantly correlate to incontinence reductions in children with OAB. The majority of children with OAB will experience increased incontinence and/or micturition frequency. However, the complexities of the syndrome make assessing separate endpoints challenging in clinical trials. Therefore, a composite endpoint, such as MVV, would be an appropriate measure for both symptoms and could reduce the number of patients required in trials to elicit clinically meaningful data, particularly important when studying a paediatric population. Prospective studies containing a large enough patient population would be helpful to further investigate these correlations in paediatric and adult patients with OAB. Given its importance as a primary endpoint, we suggest the inclusion of MVV in the OAB definition.

## Abbreviations

DMaxVV: Daytime maximum volume voided/micturition
EoS: End of study
EoT: End of treatment
MVV: Mean volume voided
OAB: Overactive bladder
SD: Standard deviation
